# Automating methods for estimating metabolite volatility

**DOI:** 10.3389/fmicb.2023.1267234

**Published:** 2023-12-14

**Authors:** Laura K. Meredith, S. Marshall Ledford, Kristina Riemer, Parker Geffre, Kelsey Graves, Linnea K. Hernandez, David LeBauer, Malak M. Tfaily, Jordan Krechmer

**Affiliations:** ^1^School of Natural Resources and the Environment, University of Arizona, Tucson, AZ, United States; ^2^BIO5 Institute, University of Arizona, Tucson, AZ, United States; ^3^Genetics Graduate Interdisciplinary Program, University of Arizona, Tucson, AZ, United States; ^4^Arizona Experiment Station, University of Arizona, Tucson, AZ, United States; ^5^Department of Environmental Science, University of Arizona, Tucson, AZ, United States; ^6^Aerodyne Research, Inc., Billerica, MA, United States

**Keywords:** bioinformatics, chemoinformatics, metabolic database, VOCs, volatile metabolite, volatility

## Abstract

The volatility of metabolites can influence their biological roles and inform optimal methods for their detection. Yet, volatility information is not readily available for the large number of described metabolites, limiting the exploration of volatility as a fundamental trait of metabolites. Here, we adapted methods to estimate vapor pressure from the functional group composition of individual molecules (SIMPOL.1) to predict the gas-phase partitioning of compounds in different environments. We implemented these methods in a new open pipeline called *volcalc* that uses chemoinformatic tools to automate these volatility estimates for all metabolites in an extensive and continuously updated pathway database: the Kyoto Encyclopedia of Genes and Genomes (KEGG) that connects metabolites, organisms, and reactions. We first benchmark the automated pipeline against a manually curated data set and show that the same category of volatility (e.g., nonvolatile, low, moderate, high) is predicted for 93% of compounds. We then demonstrate how *volcalc* might be used to generate and test hypotheses about the role of volatility in biological systems and organisms. Specifically, we estimate that 3.4 and 26.6% of compounds in KEGG have high volatility depending on the environment (soil vs. clean atmosphere, respectively) and that a core set of volatiles is shared among all domains of life (30%) with the largest proportion of kingdom-specific volatiles identified in bacteria. With *volcalc*, we lay a foundation for uncovering the role of the volatilome using an approach that is easily integrated with other bioinformatic pipelines and can be continually refined to consider additional dimensions to volatility. The *volcalc* package is an accessible tool to help design and test hypotheses on volatile metabolites and their unique roles in biological systems.

## 1 Introduction

Life is based on metabolic processes that produce and transform compounds and some of these metabolites are volatile under typical environmental conditions. Volatile compounds tend to remain in the gas phase rather than condense, and this tendency is represented by their higher vapor pressure (Finlayson-Pitts and Pitts, 1986). While the volatility of some metabolites may be widely appreciated [e.g., carbon dioxide (CO_2_), isoprene (C_5_H_8_)], volatility information is not readily available for most metabolites and is often overlooked (Meredith and Tfaily, 2022). The inability to broadly predict the volatility of metabolites cycled by organisms is problematic because volatility is a key characteristic that influences how metabolites behave. Gas-phase metabolites diffuse more readily across cellular membranes and between interacting organisms than their condensed counterparts. Therefore, volatile metabolites influence wider regions, for example by extending the rhizosphere zone beyond local rhizodeposits (de la Porte et al., 2020; Raza et al., 2021) and connecting aquatic to terrestrial ecosystems (Fink, 2007), which facilitates important ecological roles in signaling, defense, and other organismal interactions (Bennett et al., 2012; Pierik et al., 2014). In addition, detecting the suite of volatile metabolites in a system (i.e., the “volatilome”) may require alternative sampling and analytical approaches from standard metabolomics pipelines that are biased against volatiles (Rowan, 2011; Honeker et al., 2021; Meredith and Tfaily, 2022). Inclusion of volatility as a fundamental trait of metabolites can help better account for the roles of gas-phase metabolites and improve their detection.

We currently lack tools to broadly predict metabolite volatility, and current databases of volatile metabolites face some limitations. Volatile compound databases and lists have been compiled from literature reports (Peñuelas et al., 2014; Abdullah et al., 2015; Lemfack et al., 2018; Karim et al., 2020; Yáñez-Serrano et al., 2021). Most focus on volatile organic compounds (VOCs) and only variably include volatile inorganic compounds [e.g., CO_2_, carbon disulfide (CS_2_), hydrogen (H_2_), carbonyl sulfide (OCS)] although they share intimate metabolic connections with organic compounds. The microbial VOC database (mVOC 3.0) contains reports of >2,000 bona fide volatile metabolites released by >1,000 bacteria and fungi aggregated from the literature (Lemfack et al., 2018). The database has been used to explore the diversity of microbial volatiles and their specificity to selected taxonomic groups (Schenkel et al., 2015). Still, these databases and lists have some limitations for broad application. For example, metabolite volatility information is included in the mVOC 3.0 database but requires searching by individual compounds via a web interface. In general, databases collating reports of volatile compound emissions are specific to the cultivated organisms, growth conditions, and detection approaches. Moreover, most databases contain information on metabolite production but not consumption, despite the prevalence of volatile compound uptake in biological systems such as soil (Rinnan and Albers, 2020). In contrast to these volatile-specific databases, extensive databases exist that are constantly updated for metabolites, regardless of volatility. For example, the Kyoto Encyclopedia of Genes and Genomes (KEGG) (Kanehisa and Goto, 2000) contains vast numbers of compounds related to biological systems (>19,000 in KEGG) that are interconnected via metabolic maps. The KEGG database represents a cornerstone for inference into the roles of metabolites, genes, pathways, and organisms in biological systems. For example, multi-omics datasets (e.g., metabolomics, metagenomics) have been linked via KEGG to describe the sensitivity of soil and rhizosphere microbial metabolism to drought (Honeker et al., 2022; Hildebrand et al., 2023), including enhanced carbon allocation to specific volatile metabolites measured in the gas phase (i.e., acetone, acetate, and diacetyl) (Honeker et al., 2023). Moreover, KEGG contains pathways that include known volatile compounds (e.g., monoterpenes in map00902 Monoterpenoid biosynthesis; methane and methanol in map00680 Methane metabolism). If the volatility of compounds in extensive metabolite databases like KEGG could be predicted, it would provide new tools to systematically evaluate the prevalence and role of volatile metabolites.

Chemoinformatic resources exist to link metabolites to their molecular structures, creating an opportunity to build pipelines to estimate metabolite volatility from molecular parameters. Specifically, these quantitative structure-property relationship (QSPR) models can estimate the impact of various functional groups in a molecule on vapor pressure. For example, the SIMPOL.1 method estimates vapor pressure based on the size of the carbon backbone and the combined impact of functional groups (e.g., rings, hydroxyl groups, amines) (Pankow and Asher, 2008). This approach has been used to predict the vapor pressure of the complex VOC chemistry in the atmosphere (Barley and McFiggans, 2010; O’Meara et al., 2014) wherein primary VOC emissions chemically evolve (age) through diverse reactions to produce a wide array of products with varying vapor pressure. The partitioning of these compounds to the gas phase depends on their vapor pressure and also the abundance of nearby condensed organic compounds (e.g., atmospheric aerosols) (Donahue et al., 2006). We recently adapted this approach to calculate the relative volatility of metabolites across several metabolic pathways through manual evaluation of molecular carbon content and functional groups (Honeker et al., 2021; Meredith and Tfaily, 2022). However, manual evaluation of the functional group profiles of individual molecules is time consuming and infeasible for estimating volatility for the tens of thousands of compounds present in pathway databases.

Here, we move past these barriers and present *volcalc*—an automated, systematic, and scalable chemoinformatics pipeline to predict metabolite volatility from critical parameters in molecular structure files associated with every compound in the extensive KEGG database of biological pathways. With this pipeline, we aimed to automate metabolite volatility prediction to eliminate the time and variability of manually evaluating individual molecular functional group profiles, providing a more direct means for integrating metabolite volatility with known metabolic pathways, organisms, and genes. First, we adapted the volatility calculations by updating functional group terms to reflect those commonly found in metabolites and estimated volatility in reference to the atmosphere. We then created a R package, *volcalc*, to estimate volatility for individual chemical compounds or all compounds within a pathway and benchmarked its performance against a custom database of manually calculated metabolite volatility. After improving and validating the method, we demonstrate how the volatility estimation pipeline can be used to generate and test biological questions including: (1) How prevalent are volatile metabolites? (2) How variable is volatility across different pathways? and (3) Which volatile metabolites are shared or unique across specific organisms? These examples demonstrate the practicality of this scalable, open-access pipeline, which can seamlessly integrate into various bioinformatic workflows. This pipeline opens up new opportunities to understand metabolite volatility as an important characteristic that influences the behavior of biological systems and the detectability of the processes therein.

## 2 Methods

### 2.1 Metabolite volatility calculation

We estimated metabolite volatility by adapting tools that predict vapor pressure (Pankow and Asher, 2008) and estimate the partitioning of atmospheric chemicals between the gaseous and condensed phases (Donahue et al., 2006), building on the approach we first described in Honeker et al. (2021). Detailed discussions of properties of and factors affecting volatility are described elsewhere in gas-particle partitioning literature (Hilal et al., 2003; Nannoolal et al., 2008; Compernolle et al., 2011; Tang et al., 2019). Here, we estimate differences in the relative volatility between biologically-relevant molecules under standard conditions (i.e., temperature and pressure) against their tendency to condense on dry sorbent material.

For each metabolite, we calculate the vapor pressure (P; atm) using the SIMPOL.1 method (Pankow and Asher, 2008), which determines overall compound volatility from the relative contribution of common functional groups using the following equation:

$$l\,o\,g_{10}\,P=b_{0}+\sum_{k}v_{k}\,b_{k}$$

for functional groups *k* = 1, 2, 3…, where *b*_0_ is a constant, *b*_*k*_ is the functional group contribution term for group *k*, and *v* is the number of groups of type *k* in the compound. In this calculation, each vapor pressure estimate starts with a constant value (*b*_0_ = 1.79). This estimate is then proportionally reduced by the number of carbon atoms (*v*_1_) in the compound in question, representing the tendency for volatility to decrease with overall molecular size (each carbon atom decreases the value by *b*_1_ = −0.438). The estimated vapor pressure is further modified based on the functionalization of the compound, where additive contributions of molecular functional groups (*k* = 2, 3, 4…) such as hydroxyl (-OH), carboxylic acid (-C[O]OH), ketone (O = O), carbon double bond (C = C), and aromatic ring, which each decrease *P* to a different degree (Table 1) based primarily on (Pankow and Asher, 2008). Some functional groups strongly reduce volatility (e.g., carboxylic acid (-C[O]OH), *b*_10_ = −3.58), while others have intermediate [e.g., ketone (O = O), *b*_9_ = −0.935] or minor impacts [e.g., carbon double bond (C = C), *b*_5_ = −0.1], and one group may slightly enhance volatility (i.e., nitrophenol, *b*_5_ = 0.0432). Contribution terms were missing in Pankow and Asher (2008) for functional groups commonly found in metabolites such as phosphate groups, which play critical biological roles (e.g., energy transfer, protein activation, membranes). We assumed similar contribution terms to functional groups with related chemical profiles and added nine new contribution terms to the calculation based on similarities in molecular structure to known terms (see term assumption in Table 1). We did not encode contributions from seven groups described by Pankow and Asher (2008) either because of their complexity to identify (e.g., C = C-C = O in non-aromatic ring, *b*_6_) or their limited relevance to KEGG metabolic pathways (e.g., carbonylperoxynitrate, *b*_25_).

**TABLE 1:** Functional groups included in volatility estimation including inclusion in manual or automated databases, assumed contribution term, and source of contribution term.

Functional group	Structure representation example	In manual database	Automated count method (specific function)	Contribution term (*b*_*k*_)	Term source or assumption	*k*
Carbon number	C_x_	Yes	ChemmineR (atom count)	−0.438	Pankow and Asher	1
Aromatic ring	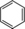	Yes	ChemmineR (rings)	−0.675	Pankow and Asher	3
Non-aromatic ring	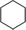	Yes	ChemmineR (rings)	−0.0104	Pankow and Asher	4
Carbon double bond	C = C	Yes	ChemmineR (conMA)	−0.105	Pankow and Asher	5
Hydroxyl group	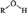	Yes	ChemmineR (groups)	−2.23	Pankow and Asher	7
Aldehyde	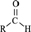	Yes	ChemmineR (groups)	−1.35	Pankow and Asher	8
Ketone	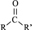	Yes	ChemmineR (groups)	−0.935	Pankow and Asher	9
Carboxylic acids	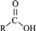	Yes	ChemmineR (groups)	−3.58	Pankow and Asher	10
Ester	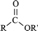	Yes	ChemmineR (groups)	−1.20	Pankow and Asher	11
Ether	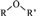	No	SMARTS and ChemmineR (smartsSearchOB)	−0.718	Pankow and Asher	12
Ether (non-aromatic/alicyclic)	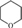	Yes	None	−0.683	Pankow and Asher	13
Ether (aromatic)	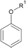	Yes	None	−1.03	Pankow and Asher	14
Nitrate	R – ONO2	Yes	SMARTS and ChemmineR (smartsSearchOB)	−2.23	Pankow and Asher	15
Nitro	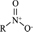	Yes	SMARTS and ChemmineR (smartsSearchOB)	−2.15	Pankow and Asher	16
Phenol	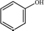	Yes	SMARTS and ChemmineR (smartsSearchOB)	−2.14	Pankow and Asher	17
Amine primary	R − NH_2_	Yes	ChemmineR (groups)	−1.03	Pankow and Asher	18
Amine secondary	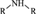	Yes	ChemmineR (groups)	−0.849	Pankow and Asher	19
Amine tertiary	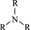	Yes	ChemmineR (groups)	−0.608	Pankow and Asher	20
Amine aromatic	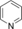	Yes	None	−1.61	Pankow and Asher	21
Amide	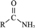	No	SMARTS and ChemmineR (smartsSearchOB)	−2.23	Same as nitrate; Rationale 1	22
Peroxide	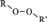	Yes	SMARTS and ChemmineR (smartsSearchOB)	−0.368	Pankow and Asher	26
Hydroperoxide	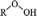	Yes	None	−2.48	Pankow and Asher	27
Nitrophenol	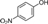	Yes	None	0.0432	Pankow and Asher	29
Nitroester	R-ONO2	Yes	None	−2.67	Pankow and Asher	30
Phosphoric acid	H_3_PO_4_	No	SMARTS and ChemmineR (smartsSearchOB)	−2.23	Same as nitrate; Rationale 1	
Phosphoric ester	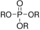	No	SMARTS and ChemmineR (smartsSearchOB)	−2.23	Same as nitrate; Rationale 1	
Phosphate	R – PO_4_	No	None	−2.23	Same as nitrate; Rationale 1	
Sulfate	R – SO_4_	No	SMARTS and ChemmineR (smartsSearchOB)	−2.23	Same as nitrate; Rationale 2	
Sulfonate	R – O_3_S	No	SMARTS and ChemmineR (smartsSearchOB)	−2.23	Same as nitrate; Rationale 2	
Thiol	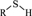	No	SMARTS and ChemmineR (smartsSearchOB)	−2.23	Same as hydroxyl group: Rationale 3	
Carbothioester	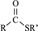	No	SMARTS and ChemmineR (smartsSearchOB)	−1.20	Same as ester; Rationale 3	

Rationale 1: Similarities in the molecular structure between group 15 atoms (P and N) may lead to similar relative contribution terms as documented in other structure-activity relationship models such as (Nannoolal et al., 2008). Rationale 2: Similarities in the molecular structure between heteroatoms (S and N) and resonant oxygen bonds may lead to similar relative contribution terms. Rationale 3: Similarity in the contributions of the same functional groups bonded to different atoms (C and S).

While the SIMPOL.1 approach has been estimated to have an error approximately one order of magnitude in vapor pressure in either direction (Barley and McFiggans, 2010), it only requires the list of functional groups in a molecule but not full structural detail of the molecule. This makes it relatively easy to compute vapor pressure compared to full structural methods and pairs well with high resolution mass spectrometer data, which can obtain elemental formulas but not structural information.

We used the vapor pressure (P) estimates to predict the volatility of metabolites by comparing their relative tendency to partition to the gas *vs.* condensed phase. Compound partitioning (*ξ*) to the gas phase increases with saturation vapor pressure (C*), which is by convention converted to mass-based saturation vapor concentration (μg m^–3^) and accounts for molecular mass. Specifically, we calculated C* from P using the ideal gas law [*log*_10_C* = *log*_10_(*PM/RT*), where M is molecular mass, R the universal gas constant, and T temperature] (O’Meara et al., 2014), without applying modifications for the intermolecular interactions of non-ideal compounds (i.e., van der Waal equation) that are relatively minor under ambient pressure (few percent) compared to variations in vapor pressure [e.g., 14 of orders of magnitude (Pankow and Asher, 2008)]. In the atmosphere, the degree that a compound partitions to the gas phase [*ξ*_*i*_ = 1/(1+C_i_*/C_*Total*_)] depends on the total availability of condensed-phase organic molecules (e.g., total aerosol; C_*Total*_) (Donahue et al., 2006). As a result, the same compound will appear less “volatile” in a polluted atmosphere with greater partitioning on high aerosol loadings.

Here, we calculate a relative volatility index (RVI) using log_10_C* (RVI = log_10_C*) as the volatility scale, with the understanding that the ultimate gas phase partitioning will differ between environments, even in the same ecosystem component (e.g., gas-filled soil pores *vs.* soil-atmosphere interface). The advantage of evaluating RVI is that it can be used to compare metabolite volatilities relative to one another irrespective of the environment. Thresholds for volatility can be defined based on gas-phase partitioning in different environments. In a clean atmosphere, lower thresholds for low, moderate, and high volatility are on the order C* = 0.01 μg m^–3^, 1 μg m^–3^, and 100 μg m^–3^, respectively, or more conveniently on a log scale: log_10_C* = −2, 0, and 2. The partitioning of compounds between condensed organic matter and gas phases will differ in other environments. For example, the volatility indices used here are derived from Donahue et al. (2006) for a clean atmosphere with relatively low concentrations of organic particulate matter available for partitioning (∼ 1 μg m-3). A polluted atmosphere such as a highway with high particulate concentrations (e.g. cooled fresh exhaust emissions) would have greater amounts of organic particulates, which would shift the RVI so that high and moderate volatility thresholds would be at 2 and 4, respectively (versus clean air at 0 and 2) (Donahue et al., 2006). These thresholds can be adapted for the environment of interest, and here we integrated these approaches to estimate vapor pressure and gas-phase partitioning to predict the volatility (RVI) of metabolites relative to the atmosphere.

First, we used this approach to manually calculate volatility of metabolites and generate a benchmarking database (Section 2.2). Second, we embedded the approach in a chemoinformatics pipeline to automate the recovery of metabolite volatility of compounds in the KEGG database (Kanehisa and Goto, 2000) and validated that approach (Section 2.3).

### 2.2 Manually curated metabolite volatility database

We constructed a database of individually calculated metabolite volatilities to evaluate the prevalence of volatile metabolites in specific pathways (Honeker et al., 2021; Meredith and Tfaily, 2022). In total, we used the volatility calculation approach to determine RVI for 474 compounds by evaluating individual molecular structures. The metabolites were drawn from nine metabolic pathways from the KEGG (00130, 00290, 00300, 00361, 00620, 00623, 00640, 00643, 00902, and 00904) (Supplementary Table 1). We focused primarily on metabolic pathways that we expected would have a high proportion of volatile metabolites (e.g., monoterpenoid biosynthesis, diterpenoid biosynthesis, toluene degradation). Compounds in the database (Supplementary Table 2) ranged in mass from 31.0571 Da (methylamine; C00218) to 1193.9458 Da (UDP-N-acetylmuramoyl-L-alanyl-D-glutamyl-6-carboxy-L-lysyl-D-alanyl-D-alanine; C04882) and in RVI from +9.73 (propane, C3 alkane; C20783) to −33.69 (isomaltose C24 sugar; C21659), excluding six compounds with inflated RVI due to manual calculation errors that overlooked n monomer repeats (C00828, C05819, C05847), multiple functional groups (C04877, C04882), and undefined R groups (C05535).

### 2.3 Development and improvement of automated volatility pipeline

**FIGURE 1:**
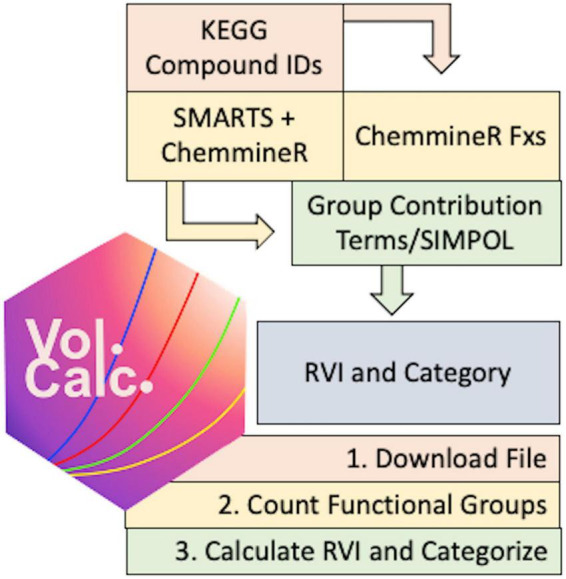
Conceptual flow of the *volcalc* pipeline.

To automate metabolite volatility predictions we built a chemoinformatic pipeline centered on the KEGG metabolic database (Kanehisa and Goto, 2000), which contains 19,048 compounds and 562 pathway maps linked to 25,555 KEGG orthology (KO) groups and 8,630 KEGG organisms (statistics as of 14 Feb 2023). As an overview, the pipeline starts from KEGG compound(s) or pathway(s), determines the molecular structure and counts functional groups, predicts the vapor pressure using the group contribution information, and calculates the RVI and volatility category for each compound (Figure 1).

We created a package in the R programming language called *volcalc* that can automatically estimate volatility for either a single chemical compound available in KEGG or for all of the compounds in a metabolic pathway in KEGG. The package is available for use at https://meredith-lab.github.io/volcalc/ and is actively under development. We used *volcalc* version 1.0.2 (Riemer and Scott, 2023) for all development and integration in this paper. The three steps to estimate volatility are analogous to the manual computation approach (Section 2.2): (1) a file that describes the molecular structure of each compound is downloaded, (2) the functional groups relevant to volatility are counted from the file, and (3) the functional groups counts are used to calculate volatility for the compound using the SIMPOL.1 method (Pankow and Asher, 2008).

The compound files are downloaded as .mol files from the KEGG database using tools from the open source KEGGREST R package (Tenenbaum, 2021). These .mol files contain consistently structured information about the composition and spatial structure of chemical compounds. The user inputs a KEGG ID for a compound or pathway to obtain these files.

The *volcalc* R package determines counts for 24 functional groups for each compound (Table 1). All of these functional groups are counted using functions in the ChemmineR R package (Cao et al., 2008) with the functions specified in Table 1. For 12 functional groups, we had to determine their corresponding SMARTS strings and, in combination with a ChemmineR function, count groups from compound .mol files. SMARTS is a language used to describe compound substructures, and we retrieved our needed strings from the Daylight Chemical Information Systems (Laguna Niguel, CA).^1^ We could not determine functional group counts for seven functional groups (Table 1) because they were unidentifiable by ChemmineR and have no readily available SMARTS string template, though there were no occurrences of three of these groups (hydroperoxide, nitrophenol, nitroester) in the manually counted database, and counts for these groups were set to zero. Compound mass came from the KEGG database (molecular weight). From differences in volatility between the automated and manual approaches, we discovered that some functional groups were double counted (e.g., a single aromatic ring being counted twice as a non-aromatic and an aromatic ring), and we assigned rules to (Table 2) correct functional group counts accordingly.

**TABLE 2:** Key rules for adjusting functional groups counts with ChemmineR output.

Case	Rule	Justification
Rings	Subtract aromatic ring from ring	Similar functional structures were being counted as both a ring and aromatic ring. Aromatic ring counts were more accurate, so those are counted in favor
Additional functional groups on a functionalized ring (e.g., -OH groups on a phenol ring)	If there’s more than one present, divide number by two and subtract by (hydroxyl - 1)	Avoid double counting of phenols and hydroxyl groups
Hydroxyl groups	Subtract phenol from hydroxyl group	Avoid double counting of phenols and hydroxyl groups
Carbon double bonds	Subtract aromatic rings times 3 from carbon double bonds	Three double bonds of aromatic rings were being counted as carbon double bonds
Phosphoric acids/esters	Subtract phosphoric ester from phosphoric acid	Similar functional structures were being counted as both phosphoric acid and phosphoric ester. Because they have same coefficient, count only one of the two

Functional group counts and mass were used to determine volatility for each compound with the SIMPOL.1 method (Pankow and Asher, 2008) (Section 2.1) and contribution terms for each functional group (Table 2). We included ten functional groups in the automated method that were not in the manual database (sources for the contribution terms given in Table 2). Each compound was also assigned a category for volatility using the clean atmosphere as the environmental context. Thresholds for these categories were as follows: nonvolatile (<−2), low volatility (−2–0), moderate volatility (0–+2), and high volatility (>+2).

The methods in the *volcalc* R package were developed iteratively, by comparing volatility values and categories estimated using the automated values for the 474 compounds to those in the manual database. The most substantial improvements in the pipeline were adding in SMARTS strings to count functional groups, adjusting overcounting from the ChemmineR functions for some functional types (Table 2), and adding additional functional groups that were not counted in the manual database (Table 1). These comparisons are available in Supplementary Table 3.

The R package is intended to provide an automated and reproducible method for efficiently and accurately predicting volatilities of individual compounds, as well as compounds associated with a specific pathway. This package includes a test suite and documentation.

### 2.4 Integration with KEGG organisms, genes, and KOs and data analysis

To demonstrate the potential to integrate *volcalc* with other KEGG datasets, we predicted the volatility of each compound in the KEGG database (Supplementary Data Sheet 1) and generated volatility profiles for each KEGG pathway (Supplementary Data Sheet 2) and organism. We collated a database of all KEGG orthologs (KOs), enzymes, reactions, and compounds (as of 14 Feb 2023) into a single database (DOI: 10.25422/azu.data.24446770). We used the R package KEGGREST to create a list of all compounds in the KEGG database and the R volcalc package function *calc_vol* to predict the volatility for each compound. We used KEGGREST to link each KO to its related enzymes, reactions, and compounds and merged these datasets to generate a database of every KO with an associated enzyme, each reaction those enzymes participate in, each compound listed in those reactions, and *volcalc*-derived information regarding the volatility of those compounds. To assess organism-level associations with volatile compounds, we used KEGGREST to generate a list of all KEGG organisms and the list of KOs associated with each organism, thereby generating unique metabolic profiles for each organism. We filtered this list against the database produced above, creating a list of KEGG organisms, their functional gene content, the compounds associated with those genes, and the volatility of those compounds. To evaluate high-quality genomes that capture the diversity of species in KEGG, we identified volatile-related genes on a subset of 973 KEGG organisms from the 975 chosen in Schulz and Almaas (2020) (two genomes were not accessible through KEGGREST). We categorized the organismal distribution of each gene-linked compound based on its associations with at least one organism in each kingdom. We pulled all pathway maps in KEGG and their associated compounds using KEGGREST and filtered these against the original compound dataset to generate a list of all pathways, associated compounds, and their volatility information. We performed all statistics in Rstudio.

## 3 Results

### 3.1 Pipeline performance benchmarked against manual database

**FIGURE 2:**
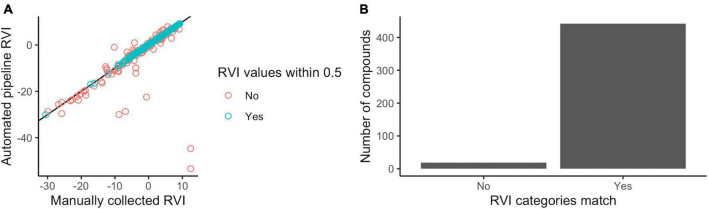
Automated *volcalc* pipeline performance benchmarked against manual calculations. **(A)** Quantitative predictions of RVI were similar (blue), i.e., within 0.5 units, between the automated and manual method and were not statistically different from each other (Wilcoxon test, *p*-value = 0.085), despite some mismatches (red). Errors in manual RVI estimation of five compounds (C04877, C04882, C00512, C09819, and C09820) was the cause of the largest outliers. **(B)** The majority of compounds were predicted to belong to the same volatility category (low, moderate, and high) by both approaches.

The automated pipeline produced RVI estimates for 98% (461 of 470) of compounds in the manual database. RVI estimates could not be determined for 9 compounds because they lacked a molecular mass in the KEGG database due to “n” monomer repeats and undefined “R” groups. The automated pipeline predicted compound RVIs that were identical (14%; 65 out of 461 compounds) or similar (75% within 0.5 units–one quarter of a 2-unit volatility category bin; 344 out of 461 compounds) to the manual database, and generally the two methods agreed, especially for volatile compounds (Figure 2A).

Differences in RVI estimates between the two methods were due to various differences in functional group counts. For the 24 functional groups that were counted by both methods (Table 1), >50 compounds (11%) differed in their functional group counts for rings, carbon double bonds, hydroxyl groups, or aromatic rings. The compounds with the greatest difference in RVI estimates had multiple differences in functional group counts. For example, the 49 compounds that had differences in RVI greater than 1 had different counts for 21 different functional groups, and the five compounds with RVI differences greater than 10 each had differences in nine or more functional groups.

We used these mismatches to diagnose and correct issues in the automated pipeline and its key calculation rules (Table 2), but also identified cases of human errors in the manual calculation database such as inconsistent avoidance of functional group double counting (e.g., ring and aromatic ring) or overlooking molecular components that are abbreviated in KEGG (e.g., acetyl-CoA groups) that led to the biggest mismatches (Figure 2A). Direct comparison between the two methods was challenging because they did not account for all the same functional groups. Six groups were not counted in the automated pipeline because they lacked a method for detection (Table 1), but only three of these groups were actually present in compounds in the comparative database (i.e., alicyclic ethers, aromatic amines, and aromatic ethers). We added nine additional functional groups to the automated pipeline that were not accounted for in the initial methods underlying the manual approach, but only six of these functional groups (i.e., ethers, amines, phosphoric esters, amides, carbothioesters, phosphoric acids) were actually present in the compounds. Importantly, despite these differences, the two methods resulted in assignment of the same volatility categories (non, low, moderate, and high) for nearly all compounds (96%; 442 of 461 compounds) (Figure 2B).

### 3.2 New capabilities to assess trends in volatility across and beyond the KEGG database

**FIGURE 3:**
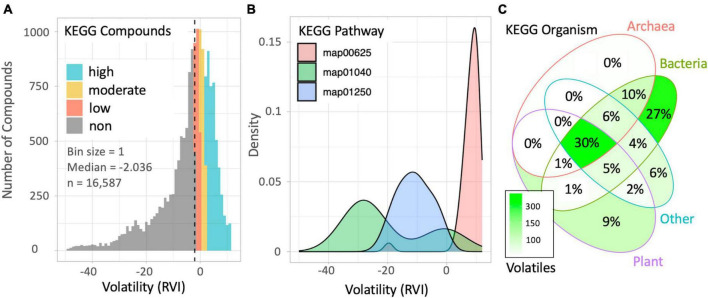
Patterns in volatility among KEGG compounds, pathways, and organisms revealed by the *volcalc* pipeline. **(A)** Counts for all compounds with estimated volatility in the KEGG database binned by RVI. **(B)** Distribution of RVI values for compounds in three specific KEGG pathways: (map01040) biosynthesis of unsaturated fatty acids; n total = 74, n volatility calculated = 69, median RVI = –27; (map01250) biosynthesis of nucleotide sugars; n total = 200, n volatility calculated = 200, median RVI = –11; (map00625) chloroalkane and chloroalkene degradation; n total = 43, n volatility calculated = 43, median RVI = 8.8. **(C)** Distribution of volatile metabolism across a subset of 973 KEGG genomes. Color indicates the number of highly volatile compounds (total = 1,124) associated with at least one gene in an organism belonging to each kingdom group. Percentage represents the fraction of volatile compounds that belong in each grouping.

We evaluated the prominence of VOCs in the KEGG database to demonstrate the potential of the *volcalc* biochemoinformatic pipeline for data mining and hypothesis generation. We successfully calculated the RVI of 16,587 of the 19,119 compounds (∼87%) in KEGG using the command *calc_vol* in the *volcalc* package. Those that were unable to be calculated included: those with incomplete data such as a missing mass or formula (e.g., C00028, hydrogen acceptor), an “n” subscript, or a nonspecific “R” group (e.g., CH_3_OR, primary alcohol). We discovered that the distribution of calculable metabolite volatility in KEGG was largely nonvolatile (49.8%; *n* = 8,345), but still with significant proportions of low (12.4%; *n* = 2,052), moderate (10.7%; *n* = 1,778) and high (26.6%; *n* = 4,412) volatility based on RVI cutoffs for the clean atmosphere (Figure 3A).

We evaluated the distribution of volatility across the compounds in KEGG pathways. For each pathway, we determined the total number of compounds, percentage of compounds with a calculated RVI, and the median compound RVI for each pathway. Of the 449 pathways containing compounds in KEGG, the median percentage of compounds that *volcalc* returned a RVI value was ∼83%. Thirty-two of the pathways had no compounds with a calculable volatility. These pathways were small (containing two compounds on average) and highly specific. For example, map01521 (EGFR tyrosine kinase inhibitor resistance) only contains the compounds diacylglycerol and phosphatidylinositol-3,4,5-trisphosphate and both contain a nonspecific “R” functional group. We calculated the volatility of 100% of compounds in 138 pathways. These included larger pathways, such as map01250 (biosynthesis of nucleotide sugars), which contains 200 compounds. To exemplify variability in the distribution of compound RVIs within different pathways, we compared three pathway maps representing a majority of volatile to nonvolatile distributions of compound RVIs (Figure 3B).

We used KEGG’s features as an “encyclopedia of genes and genomes” to draw associations between organisms and volatile metabolites, revealing variability in the distribution of volatiles across all life (Figure 3C; Supplementary Table 4). Of the 1,124 volatile metabolites identified linked to the subset collection of 973 organisms in KEGG, 30% were associated with at least one gene across kingdoms. This highlights a set of highly conserved volatiles which may be associated with core biotic functions. Seventy-eight volatiles were found in at least 90% of organisms and included compounds such as ammonia, water, and formate. Conversely, 9% of KEGG volatiles were found to be only associated with plants, while prokaryotes are tied to 37% of unique KEGG volatile compounds, mostly only within bacteria (27%). Overall, more than half (62%) of the volatiles identified in this subset were associated with less than 10% of organisms.

## 4 Discussion

### 4.1 New capabilities with volcalc

We present *volcalc*—a new tool to systematically estimate the relative volatility of metabolites—that can be applied to single compounds, all metabolites within a pathway, and even the entire collection of metabolites (>19,000) in the KEGG database. The relatively straightforward approach helps illustrate key factors influencing metabolite volatility: functionality of the compound and the environment in which it exists (Pankow and Asher, 2008). The method itself can help build an intuitive sense for how different functional groups may influence metabolite volatility, as each contribution term “taxes” the volatility to a different degree (Table 1), including newly added functional groups that are uncommonly observed in atmospheric chemistry but are common in metabolites.

*Volcalc* compares metabolites and their volatility on a consistent basis (molecular structure). This may avoid discrepancies in databases that draw volatility information from a mixture of experimental and theoretical values and incomplete chemical databases that are variably linked to metabolites via chemical identifiers. This approach has shown promise in standardizing volatility even through manual calculations (Honeker et al., 2021; Meredith and Tfaily, 2022). Automating the *volcalc* pipeline overcomes the limitations of manual calculations, which are laborious and time consuming. The automated pipeline resulted in comparable accuracy to manual calculations (Figure 2), while avoiding human errors associated with large manually-calculated databases. The tool can be run on all or a few compounds in KEGG metabolic pathways or the entire database, providing flexibility and scope and adaptability for updates of the KEGG database. Moreover, KEGG compound IDs are interconvertible with other chemical identifiers (e.g., SMILES) using KEGGREST, providing a means to apply *volcalc* on compound lists originating outside of KEGG, and they can be used to pull additional chemical information from other datasets. We release *volcalc* for free use under the MIT open source license^2^ that is available to download and run in R. *Volcalc* therefore permits scalable, open, and consistent predictions of metabolite volatility that can be used to ask broad questions and generate hypotheses that could not have been easily addressed otherwise.

Using *volcalc*, we demonstrate that volatile metabolites may represent a significant fraction of cataloged metabolites. We identify >4,000 candidate high volatility metabolites, which is a similar order of magnitude to the >2,000 VOCs collated in the extensive microbial VOC database (Lemfack et al., 2018). Expanding our RVI estimates from a manual database of 8 metabolic pathways known to contain volatiles (474 non-redundant metabolites) (Meredith and Tfaily, 2022) to the entire KEGG database (>19,000 compounds), we obtain similar estimates of the percentage of volatile metabolites in the atmosphere (from 47 to 50%, respectively), revealing that volatile metabolites make up a sizeable proportion of known metabolites across the diverse pathways in life.

*Volcalc* generates volatility categories relative to the assumed environmental thresholds and the estimated RVI values, allowing the role of the local environment and its organic matter on the partitioning of metabolites to the gas phase to be considered. Critically, the partitioning of compounds between organic condensed and gas phases will differ in other environments, for example in clean versus polluted atmospheres or soils with different amounts of organic matter. Thus, the volatility thresholds can be adapted for the environment of interest. For example, soil is an important reservoir of diverse organic compounds of varying volatility (Honeker et al., 2021). While the large quantities of soil organic matter (often ∼1% g C g^–1^ soil) would drive volatiles to partition more preferentially to the condensed phase, the organic matter is occluded in aggregates and is heterogeneously distributed on surfaces, and theories linking total soil carbon to thresholds for volatility in the subsurface have not been established. We assumed thresholds in soil for low and high volatility could be as high as 4 and 8, respectively, under a scenario where only < 0.1% of the soil carbon is available for partitioning (Meredith and Tfaily, 2022), which would shift estimates for KEGG metabolite volatility to 16.5 and 3.4%, respectively of the 16,587 compounds with RVI calculations. Users of *volcalc* can adjust volatility thresholds to reflect the availability of condensed organic matter in their environments of interest and use the relative nature of the RVI estimates (continuous data) to compare compounds irrespective of environment.

Metabolic pathway maps are important resources for relating metabolites to each other in biological systems and inferring specific types of biological activity from the detection of metabolites. *Volcalc* estimates the prevalence of volatiles in pathways, revealing some that are dominated by volatile compounds, while others are strictly nonvolatile (Figure 3B), depending on the environmental conditions. Recognizing and quantifying this variability is important for predicting whether metabolites are undetected due to their absence or due to the measurement biases against volatiles (Honeker et al., 2021). Moreover, identifying pathway-dependent volatility trends can help identify where approaches targeted toward measuring volatile compounds might be particularly useful for monitoring some biological functions that may be obscured from traditional metabolomics methods. Pathways with a high proportion of volatile metabolites present an opportunity for biological monitoring using non-invasive gas-phase volatilomics approaches (Meredith and Tfaily, 2022).

*Volcalc* can be used to extrapolate volatile metabolism onto particular genes and genomes to identify trends in volatile metabolism across organisms. By linking all KEGG genes to their associated enzymes and reactions, we associated genetics and volatilomics across kingdoms. Current understanding of the genetic component of volatile metabolism is extremely limited, particularly in non-plant species. Using *volcalc*, we showed that four times more volatile metabolites were identified to be uniquely associated with prokaryotic species (37%) than those only found in plants (9%) (Figure 3C). Therefore, this gene-centric approach can be used to assess volatile metabolism distribution among species and build understanding of widespread volatile metabolism. The *volcalc* approach is complementary to volatile metabolite databases, e.g., (Lemfack et al., 2018), which provides confirmation of *volcalc*-predicted volatile-producing phenotypes under particular growth conditions but requires manual retrieval of compound information one at a time. Conversely, our approach taps into all known metabolites, pathways, and genomes in KEGG, and may better identify volatile-producing genotypes and also volatile-consuming genotypes. Nevertheless, we remain restricted to the known metabolites and pathways that have been incorporated into KEGG. Still, even with the potential biases in KEGG toward pathways, organisms, and metabolites with industrial, medical, or environmental relevance and organisms amenable to culturing (Albright and Louca, 2023), KEGG is one of the most comprehensive database of current understanding of metabolism used across the biological sciences. *Volcalc* is a useful tool for estimating the size and diversity of the volatilome in different biological systems to generate testable hypotheses that improve understanding of the roles of volatile metabolites and design studies for their detection.

### 4.2 Approximations and areas for growth

The automated *volcalc* method provides an estimation of volatility, but it has several limitations. On a practical level, the contribution terms for some functional groups found in KEGG metabolites were not defined, and our assumptions (Table 1) based on related molecular properties need to be experimentally validated. For example, we assumed contribution terms for phosphate-containing functional groups through analogy to empirically derived values for nitrate groups and similar group contribution terms in other approaches (Nannoolal et al., 2008). Few P-containing compounds have been described in volatile organic compound databases [e.g., 0–2% of compounds (Lemfack et al., 2018; Pagonis et al., 2019; Yáñez-Serrano et al., 2021)], although organic phosphorus compounds have been observed in the gas phase (Li et al., 2020) in biological systems (11% of compounds in KEGG). Moreover, contribution terms are not yet included in volcalc for all heteroatoms including halides, metals, and some nonmetals (e.g., selenium) that make up a minor fraction of metabolites. In some cases, it was difficult to definitely categorize or precisely count the functional groups of complex molecular structures (e.g., bridged rings) and interacting functional groups (e.g., phenol rings with a second hydroxyl group). These factors led to uncertainty in both the manual database and automated *volcalc* pipeline. Moreover, the manual database contained human errors and the automated pipeline had some limitations to functional group counting with the tools available (chemmineR/SMILES).

Volatility itself is not defined by a single numerical quantity, but can be described using vapor pressure, boiling point, Henry’s law constants, solubility or a combination thereof. We use the SIMPOL.1 method that estimates vapor pressure from the number and type of functional groups in a molecule to account for intermolecular forces along with molecular weight, which are primary contributing factors to volatility. This straightforward QSPR model does not take into account most higher-order interactions between complex arrangements of groups within a molecule (e.g., intramolecular hydrogen-bonding, dipole moments) or whether a molecule is charged and their possible integrated influence on intermolecular forces (IMF). Increasing IMFs tend to reduce volatility, and the SIMPOL.1 approach may overestimate volatility for compounds with these IMFs. Indeed, like other group contribution methods, SIMPOL.1 overestimates the vapor pressure of compounds with multiple hydrogen-bonding groups (O’Meara et al., 2014) such as amino acids (e.g., leucine, serine). Overall, error in the SIMPOL.1 method imparts approximately a 1-unit uncertainty in RVI in either direction. While SIMPOL.1 is a reliable and quick method for volatility estimates, there may be particular instances where alternatives may be required to estimate more accurate vapor pressures.

In *volcalc*, we use predicted vapor pressure to calculate RVI that are then used to compare to partitioning thresholds and estimate the degree to which compounds partition to the gas phase (i.e., resist condensing) in a given environment. An RVI is useful for comparing the relative propensity for partitioning between compounds, but as calculated it currently makes this comparison under standard conditions, for gas-partitioning onto dry material, and with the ideal gas law. SIMPOL.1 has the framework to project vapor pressure at non-standard temperature, however, the temperature sensitivity of group coefficients have not been defined (Pankow and Asher, 2008). The moisture content and surface interactions in different environments will also vary. Instead of using the ideal gas law, vapor pressure could be converted to concentration more accurately using the van der Waals equation, although this is a relatively minor correction under typical ambient conditions (e.g., low pressure, large volumes) and requires knowing or predicting an additional two constants per compound. Correspondingly, the likelihood of observing a compound in the gas phase will also be influenced by its solubility, equilibrium concentration above liquid (e.g., Henry’s Law constant), and the matrix-specific adsorption terms such as soil adsorption coefficients (*K*_*d*_) or the organic carbon-water partition coefficient (*K*_*oc*_). This information has been compiled, estimated, and interpreted for some of the compounds in the mVOC 3.0 database (Lemfack et al., 2018), but lacks a prediction framework analogous to SIMPOL.1 for automation here. Finally, environmental pH modifies compound charges (e.g., organic salts *vs.* acids, conjugate acid-base pairs like acetate and acetic acid) and thus their IMF. Metabolic pathways typically list only one of these compound forms, and while *volcalc* may overestimate the volatility of charged moieties, they may exist in equilibrium with their more volatile forms in the environment making them still useful harbingers for volatility. The overall importance of these terms will depend on the conditions and biological system.

Finally, we recognize that *volcalc* relies on a single database. While extensive, KEGG represents only one database and any predictions made from *volcalc* rely on the extent and accuracy of its currently available data. For example, isoprene is the most prominently produced non-methane VOC on Earth. While KEGG includes the pathway for isoprene production (often by plants), it does not include those for isoprene degradation (often by microorganisms) that can be found in other databases (Caspi et al., 2016). In addition, some data for specific compounds are incomplete. This includes those for which we could not predict volatility due to missing mass.

### 4.3 Concluding summary and future directions

While often overlooked, volatility is a characteristic of metabolites that can have pervasive impacts on their function, detectability, and influence. *Volcalc* is an easily accessible and simple method of estimating metabolite RVIs, and its power is magnified through its linkage with the KEGG database. Here, we highlight *volcalc’s* accuracy as a volatility predictor and outline its potential uses in a research context. Specifically, we illustrated how *volcalc* can help define the diversity and reach of the volatilome, inform targeted and untargeted metabolomic approaches and inference, and interrogate the specificity and conservation of volatile metabolism across organisms.

As a fast, flexible, and straightforward tool, *volcalc* can be adapted for new directions. *Volcalc* could be implemented in ‘omics pipelines that connect genes, metabolites, and organisms in data sets derived from the environment. For example, metagenomic and metatranscriptomic pipelines could categorize genes by their association to volatile metabolites to generate and test hypotheses on the role of volatiles in biological systems. Moreover, the integration of volatility predictions within metabolomic pipelines like MetaboDirect, in conjunction with the computation of additional indices such as NOSC, GFE, and aromaticity index, holds great potential to augment their ecological relevance (Ayala-Ortiz et al., 2023). This integrated approach can serve as an effective means to predict metabolite stability, fate, and decomposition pathways. The *volcalc* framework could be expanded to integrate other information from the KEGG database and to run on compounds from other chemical and biological databases [e.g., MetaCyc (Caspi et al., 2016)] or directly on lists of molecular structure files. Moreover, other properties of molecules that can be predicted from molecular structure could be included in the pipeline, e.g., estimates of metabolite solubility, charges, propensity to act as acids or bases, and polarity, to more thoroughly gauge the partitioning of metabolites in complex matrices. Along these lines, future work that experimentally validates the volatility of *volcalc* predictions will be a valuable step toward improving the tool, and other vapor-pressure estimation methods (e.g., Nannoolal et al., 2008) could be included as user options. Finally, deeper comparison of *volcalc* with volatile databases could help identify key organisms and genes that remain to be evaluated for volatile production and consumption and conversely identify volatile metabolites that are biologically produced, but lack a known or described pathway in databases.

## Data availability statement

The datasets presented in this study can be found in online repositories. The names of the repository/repositories and accession number(s) can be found in this article/Supplementary material and in the University of Arizona Research Data Repository at https://doi.org/10.25422/azu.data.24446770.

## Author contributions

LM: Conceptualization, Funding acquisition, Methodology, Supervision, Writing—original draft, Writing—review and editing. SL: Data curation, Formal analysis, Investigation, Methodology, Visualization, Writing—original draft, Writing—review and editing. KR: Data curation, Formal analysis, Investigation, Methodology, Software, Visualization, Writing—original draft. PG: Data curation, Formal analysis, Visualization, Writing—review and editing. KG: Data curation, Formal analysis, Writing—review and editing. LH: Data curation, Methodology, Supervision, Writing—review and editing. DL: Software, Writing—review and editing. MT: Conceptualization, Writing—review and editing. JK: Conceptualization, Investigation, Methodology, Writing—review and editing.

## Footnotes

1
https://www.daylight.com/

2
https://github.com/Meredith-Lab/volcalc

## Supplementary material

Supplementary Table 1 Metabolic pathways used to generate manual database.

Supplementary Table 2 Manual RVI database.

Supplementary Table 3 Automated pipeline compare to manual.

Supplementary Table 4 Compounds organisms high volatility.

Supplementary Data Sheet 1 KEGG compound volatility.

Supplementary Data Sheet 2 All Compounds in pathways list.
