# Supplementary material for: Automating methods for estimating metabolite volatility
Source: Front Microbiol. 2023 Dec 14;14:1267234. doi: 10.3389/fmicb.2023.1267234 (PMC10755872; doi:10.3389/fmicb.2023.1267234)
Supplement: Supplementary Table 1 — Metabolic pathways used to generate manual database. [file Table_1.DOCX]

***Supplementary Material***

# Supplementary Tables

| **Map** | **Pathway name** |
| --- | --- |
| map00130 | Ubiquinone and other terpenoid quinone biosynthesis |
| map00290 | Valine, Leucine and Isoleucine Biosynthesis |
| map00300 | Lysine Biosynthesis |
| map00361 | Chlorocyclo hexane & Chloro benzene Degradation |
| map00620 | Pyruvate Metabolism |
| map00623 | Toluene Degradation |
| map00640 | Propanoate Biosynthesis |
| map00902 | Biosynthesis of Monoterpenoids |
| map00904 | Diterpenoid biosynthesis |
